# A Treatise on the Decline of Manhood

**Published:** 1885-09

**Authors:** 


A TREATISE ON THE DECLINE OF MANHOOD. By A. E. Small, A. M., M. D. Duncan Bros. Chicago, 1885.

This is the third edition of this well-known little work. The subjects of which it treats are among the most important causes of disease that the physician has to encounter. Yet they are but little considered, because so deeply hidden. Dr. Small's book serves to arouse attention to these causes and therefore should be in the library of every man practicing medicine.

W. M. J.

ARNDT'S SYSTEM OF MEDICINE.

The second volume of this elaborate work has just been issued from the Hahnemann Publishing House of F. E. Bœricke. A review of it has already appeared in the pages of THE HOMŒOPATHIC PHYSICIAN.

